# Marine Sediments Hold an Untapped Potential for Novel Taxonomic and Bioactive Bacterial Diversity

**DOI:** 10.1128/mSystems.00782-20

**Published:** 2020-09-15

**Authors:** Pernille Kjersgaard Bech, Klaus Lars Lysdal, Lone Gram, Mikkel Bentzon-Tilia, Mikael Lenz Strube

**Affiliations:** a Department of Biotechnology and Biomedicine, Technical University of Denmark, Kongens Lyngby, Denmark; University of Sao Paulo

**Keywords:** antibiotics, marine microorganisms, secondary metabolites, nonribosomal peptides, polyketides, amplicon sequencing

## Abstract

Novel natural products have traditionally been sourced from culturable soil microorganisms, whereas marine sources have been less explored. The purpose of this study was to profile the microbial biosynthetic potential in coastal surface seawater and sandy sediment samples and to evaluate the feasibility of capturing this potential using traditional culturing methods. Amplicon sequencing of conserved ketosynthase (KS) and adenylation (AD) domains within polyketide synthase (PKS) and nonribosomal peptide synthetase (NRPS) genes showed that seawater and, in particular, sandy sediment had a high biosynthetic potential with 6,065 and 11,072 KS operational biosynthetic units (OBUs) and 3,292 and 5,691 AD OBUs, respectively, compared to that of four soil samples collected by Charlop-Powers et al. (Z. Charlop-Powers, C. C. Pregitzer, C. Lemetre, M. A. Ternei, et al., Proc Natl Acad Sci U S A 113:14811–14816, 2016, https://doi.org/10.1073/pnas.1615581113) with 7,067 KS and 1,629 AD OBUs. All three niches harbored unique OBUs (*P* = 0.001 for KS and *P* = 0.002 for AD by permutational multivariate analysis of variance [PERMANOVA]). The total colonial growth captured 1.9% of KS and 13.6% of AD OBUs from seawater and 2.2% KS and 12.5% AD OBUs from sediment. In a subset of bioactive isolates, only four KS OBUs and one AD OBU were recovered from whole-genome sequencing (WGS) of seven seawater-derived strains and one AD OBU from a sediment-derived strain, adding up to 0.028% of the original OBU diversity. Using a pairwise regression model of classified amplicon sequence variants (ASVs) to the species level, and OBUs, we suggest a method to estimate possible links between taxonomy and biosynthetic potential, which indicated that low abundance organisms may hold a disproportional share of the biosynthetic potential. Thus, marine microorganisms are a rich source of novel bioactive potential, which is difficult to access with traditional culturing methods.

**IMPORTANCE** Since bacterial resistance to antibiotics is developing worldwide, new antibiotics are needed. Most antibiotics discovered so far have been found in soil-dwelling bacteria, so we instead targeted marine environments as a novel source of bioactive potential. We used amplicon sequencing of bioactive gene clusters in the microbiome of coastal seawater and sandy sediments and found the bioactive potential to be comparable to, but distinct from, the bioactive potential of selected soil microbiomes. Moreover, most of this potential is not captured by culturing. Comparing the biosynthetic potential to the corresponding microbiome composition suggested that minor constituents of the microbiome likely hold a disproportionally large fraction of the biosynthesis potential.

## Abstract (precis)

Since bacterial resistance to antibiotics is developing worldwide, new antibiotics are needed. Most antibiotics discovered so far have been found in soil-dwelling bacteria, so we instead targeted marine environments as a novel source of bioactive potential. We used amplicon sequencing of bioactive gene clusters in the microbiome of coastal seawater and sandy sediments and found the bioactive potential to be comparable to, but distinct from, the bioactive potential of selected soil microbiomes. Moreover, most of this potential is not captured by culturing. Comparing the biosynthetic potential to the corresponding microbiome composition suggested that minor constituents of the microbiome likely hold a disproportionally large fraction of the biosynthesis potential.

## INTRODUCTION

Since the discovery of penicillin, microorganisms have been our most prolific source of antibiotics and other compounds with medicinal importance (1). However, the rapid development and spread of antibiotic resistance are calling for further bioprospecting efforts to find and develop novel natural products that can enable treatment of infectious diseases where the causative agent(s) has become resistant to currently available antibiotics.

Cultured environmental bacteria, especially from soil and other terrestrial environments, have been the most fruitful source of antibiotics and other bioactive natural products; however, in the past decades, very few new antibiotics have been discovered, in part due to high rediscovery rates from the continued examination of cultured bacteria. The explosion in microbial genome sequencing and the development of algorithms, such as antiSMASH (2, 3), which allows for systematic mining of genomes for specific genetic features, have shown that microbial genomes harbor a wealth of biosynthetic gene clusters (BGCs) for which the corresponding chemistry is not currently known (4). This discovery has led to the expansion of both genome sequencing of cultured microorganisms and to large-scale sequencing of environmental microbiomes to determine which niches represent rich natural product diversity that can be harvested either by improved cultivation techniques or, potentially, by cloning and heterologous expression from metagenomic libraries (5–7).

As mentioned, cultured soil bacteria belonging to the actinobacteria (8), bacilli (9), and myxobacteria (10) have been a rich source of bioactive natural products. However, the need for novel chemistry has led to exploration of other environments, and during the last 3 decades, several novel microbial secondary metabolites with clinical relevance have been isolated from marine systems (11).

Given the vastness of the marine environment with oceans constituting more than 95% of the biosphere, it is believed that novel chemistry can be bioprospected from marine environments. Moreover, the marine environmental conditions are different from those of the terrestrial zone, and indeed, more than 70% of marine natural products are not represented in terrestrial natural products (12). Initial exploration of marine natural product chemistry led to the isolation of several bioactive compounds from eukaryotic organisms, such as bryostatin and trabectedin (13, 14); however, recent genome-based studies have revealed that these compounds are produced by associated prokaryotic microorganisms (15, 16). Bioprospecting of marine microorganisms has relied on cultivation, and classical bioassay-guided fractionation has been used to isolate novel compounds from organisms such as *Salinospora* (17), *Pseudoalteromonas* (18), roseobacters (19), and *Vibrionaceae* (20).

Whole-genome sequencing (WGS) of cultured microorganisms has, as mentioned, revealed an untapped genetic potential for microbial production of bioactive compounds (21). However, the potential of uncultured microorganisms is believed to be even larger based on deep amplicon sequencing of environmental DNA (eDNA) from soil samples (22, 23). While cloning and heterologous expression from metagenomic data are possible (24–26), this is still a very difficult process, and a genome- and transcriptome-guided isolation of compounds from cultured microorganisms is likely a faster approach. However, a sequence-guided approach to select niches from where to aim cultivation efforts is an important bioprospecting strategy.

Given the many novel bioactive compounds isolated from cultured marine microorganisms, we hypothesized that marine environments harbor a great genetic potential for bioactive compound production. The purpose of the present study was to explore this by using a targeted sequence-based approach to determine the potential for production of secondary metabolites in marine microbial communities. We used an amplicon sequencing approach developed by the Brady laboratory (23) targeting conserved domains of the synth(et)ases of two of the major classes of bioactive natural products, the nonribosomal peptides (NRPs) and polyketides (PKs). We also evaluated to what extent classical culture-based bioprospecting approaches could facilitate the isolation of bacteria carrying the genetic biosynthesis potential observed by direct sequencing. Finally, to guide further targeted culture and isolation strategies, we developed a model that allowed us to compare the sequence-based biosynthesis potential with the metataxonomic profile of the samples. Previous efforts linking the two have largely been focused on mining metagenomic genomes (27) or on phylum-level taxonomic associations of functional diversity (28), which so far has been insufficient for identifying novel species, genera, or families exhibiting extensive biosynthetic repertoires.

## RESULTS

### Microbial community composition.

The microbial communities in coastal surface seawater and sandy sediments represented populations composed of 7.11 × 10^6^ ± 1.43 × 10^6^ cells ml^−1^ and 3.09 × 10^7^ ± 0.76 × 10^7^ cells g^−1^, respectively. The diversity and composition of the communities were determined by sequencing the 16S rRNA V3V4 amplicons. After processing, they contained on average 38,958 ± 29,461 and 332,883 ± 218,224 classified bacterial reads per sample (Table 1) representing 1,086 and 1,099 species (classified amplicon sequence variants [ASVs]), respectively. Despite the higher bacterial abundance in sediments, Chao1 richness estimates suggested that seawater and sediments comprised a similar number of distinct species with a mean ± standard deviation (SD) of 1,378 ± 61 species for seawater and 1,452 ± 74 species for sediments, respectively. No significant difference between the richness estimates was observed (*P* = 0.25).

**TABLE 1:** Descriptive statistics of the samples used in the study^*a*^

Sample ID^*b*^	Source	Medium	No. of V3V4 reads	No. of species	Species Chao1	KS			AD		
						No. of reads	No. of OBUs	Chao1	No. of reads	No. of OBUs	Chao1
W_	Seawater		38,958 ± 29,461	1,086	1,378 ± 61	112,548 ± 12,355	5,312	6,065 ± 81	144,340 ± 16,503	2,463	3,292 ± 109
S_	Sediment		332,883 ± 218,224	1,099	1,452 ± 74	134,393 ± 34,158	10,314	11,072 ± 70	118,985 ± 14,794	5,369	5,691 ± 43
MBA_W	Seawater	Marine broth	409,450 ± 11,805	199	304 ± 47	169,630 ± 69,150	619	1,853 ± 312	277,375 ± 63,993	329	1,972 ± 657
MBA_S	Sediment	Marine broth	263,219 ± 198,978	203	354 ± 63	119,737 ± 8,989	873	2,531 ± 302	217,702 ± 86,319	960	2,208 ± 201
MBG_W	Seawater	Marine broth	199,321 ± 191,228	149	274 ± 61	166,410 ± 69,832	1,115	4613 ± 647	129,759 ± 11,457	463	1,220 ± 183
MBG_S	Sediment	Marine broth	176,332 ± 145,515	222	424 ± 79	135,917 ± 44,099	1,782	9,248 ± 1,341	229,762 ± 42,865	1,281	4,602 ± 627
SWA_W	Seawater	Seawater	353,591 ± 204,575	245	360 ± 47	146,247 ± 25,299	757	1,273 ± 108	240,077 ± 64,379	1,002	2,457 ± 282
SWA_S	Sediment	Seawater	171,032 ± 68,737	342	845 ± 169	71,587 ± 34,631	1,43	10,915 ± 2,230	197,024 ± 30,947	774	1,788 ± 182
SWG_W	Seawater	Seawater	153,498 ± 77,025	180	330 ± 70	113,337 ± 32,470	847	2,060 ± 248	289,908 ± 73,856	1,33	3,810 ± 425
SWG_S	Sediment	Seawater	65,104 ± 52,877	184	363 ± 77	131,844 ± 5,650	1,722	7,542 ± 999	162,169 ± 103,467	690	2,484 ± 399
DFD	Soil					50,400 ± 19,622	6,966	7,067 ± 777	83,156 ± 19,511	1,59	1,629 ± 428

a Values are summarized for all samples within each sample type and given as sequence reads, observed species and KS and AD operational biosynthetic units (OBUs) and the corresponding Chao1 indices. Values are given as means ± standard deviations.

b ID, identifier.

**FIG 1:**
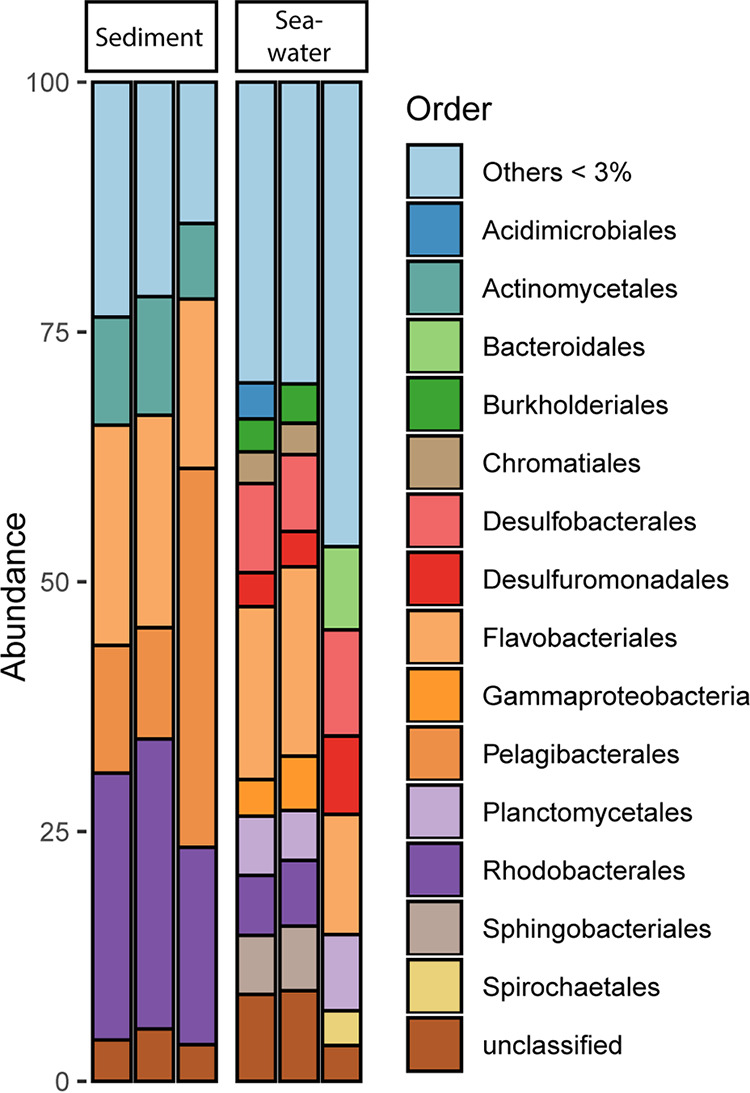
Representation at the order level of the relative taxonomic abundance of 16S rRNA V3V4 gene sequences found in each replicate of sandy sediment and seawater eDNA samples. Species assigned to orders with <3% of the relative abundance were grouped as Others.

At the time of sampling, the microbial community in the seawater was dominated by *Acidimicrobiales* (4.0 to 6.8%), *Desulfobacterales* (12.0 to 18.3%), *Flavobacteriales* (10.0 to 12.6%), *Planctomycetales* (9.2 to 18.5%), *Sphingobacteriales* (2.3 to 9.1%), and a large fraction of unclassified *Proteobacteria* (19.4 to 43.4%) containing up to 14% unclassified *Gammaproteobacteria*. The marine sediments were dominated by *Actinomycetales* (8.1 to 12.9%), *Flavobacteriales* (16.4 to 22.1%), unclassified bacteria (6.1 to 8.7%), and a large fraction of *Alphaproteobacteria* of the order *Pelagibacterales* (12.3 to 41.4%) with only one dominating species classified as the SAR11 clade, and the *Rhodobacterales* order (20.4 to 30.51%; Fig. 1).

### Biosynthesis potential of marine and soil microbiomes.

**FIG 2:**
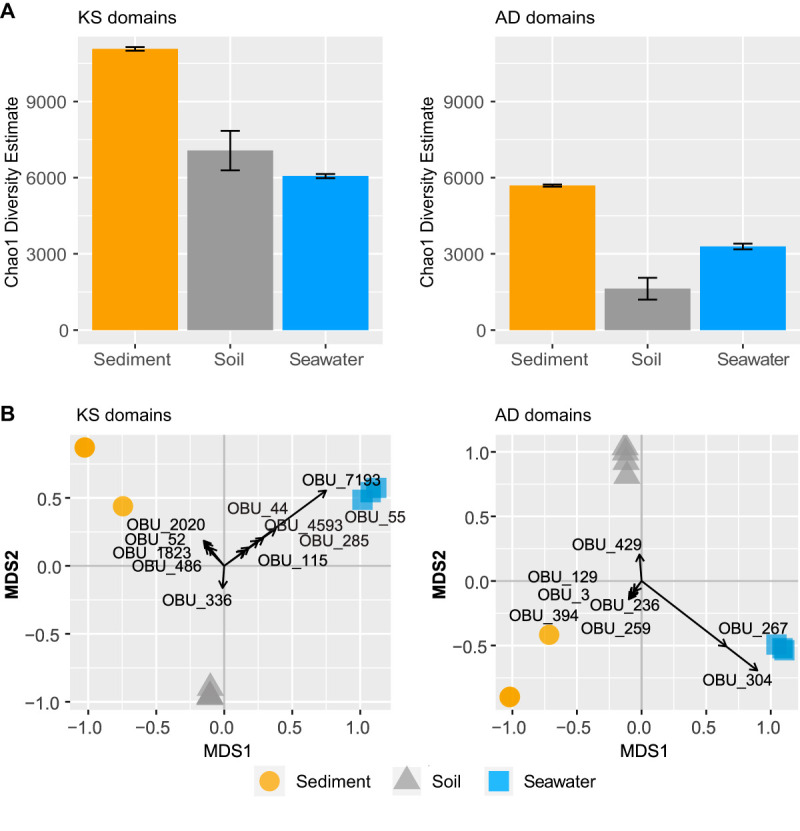
Diversity of the KS and AD OBUs found in the eDNA. (A) Chao1 richness estimates of KS and AD OBU sequences from sandy sediment (yellow circles), soil (gray triangles), and seawater (blue squares). Differences were tested with ANOVA, and if significant, followed by Tukey’s test. Different medians were denoted by different letters. (B) Multidimensional scaling (MDS) biplot of sediment (yellow circles), soil (gray triangles), and seawater (blue squares) based on the KS and AD OBU Bray-Curtis dissimilarity matrices. OBUs with absolute loading magnitudes > 0.1 are shown in the graph.

The genetic potential for production of NRPs and PKs in surface seawater samples (named “W_”) and sandy sediment samples (named “S_”) was analyzed by amplicon sequencing. We used degenerated primers to amplify the conserved ketosynthase (KS) domains and adenylation (AD) domains in polyketide synthases (PKS) and nonribosomal peptide synthetases (NRPS) from environmental DNA (eDNA). This represented the genetic diversity of microbial polyketides and nonribosomal peptides and was compared to sequences obtained from terrestrial soil samples (named “DFD”) chosen as representatives of a study of urban park soils (23). All sequences were processed with the same pipeline and clustered at 95% sequence identity, yielding 94,242 KS and 112,259 AD reads per sample on average. These represented a total of 5,312, 10,314, and 6,966 KS observed operational biosynthetic units (OBUs) and 2,463, 5,369, and 1,590 AD OBUs from seawater, sediment, and soil samples, respectively. To investigate the KS and AD production potential within marine and soil sample microbiomes, Chao1 richness estimates of KS and AD OBUs found in seawater, marine sediments, and soil samples were calculated. Thousands of KS OBUs (mean, 11,072 ± 70) and AD OBUs (mean, 5,691 ± 43) were estimated to be found in the sandy sediment samples, which relative to soil (mean for KS, 7,067 ± 776; mean for AD, 1,629 ± 428) showed a significantly higher biosynthetic potential (*P* = 0.002 for KS; *P* = 0.0003 for AD) (Fig. 2A and Table 1). The seawater communities harbored a comparable richness of KS OBUs (mean, 6,065 ± 81) to the soil samples (*P* = 0.08) but a higher richness of AD OBUs (mean, 3,292 ± 109) (*P* = 0.003).

The composition of KS and AD OBUs was analyzed by a principal coordinate ordination analysis showing that the three environments harbored distinct microbiomes in terms of their potential for biosynthesis of secondary metabolites, as both the KS and AD OBUs found within seawater, marine sediment, and soil samples clustered into distinct groups by principal coordinate analysis (Fig. 2B). This observation was further supported by a high degree of multivariate differentiation (*P* = 0.001 for KS and *P* = 0.002 for AD by permutational multivariate analysis of variance [PERMANOVA]).

### Database-driven taxonomic annotation of KS and AD OBUs.

We successfully mapped 4.5% and 12.5% of the KS and AD OBUs from soil samples to KS and AD OBU reference genomes in the MiBiG database, respectively. In contrast, only 0.3% KS and 1.5% AD OBUs from seawater and 0.3% KS and 2.6% AD OBUs from sediment were annotated by this approach. The majority of classified OBUs from soil mapped to genomes of *Streptomyces* spp., *Mycobacterium* spp., *Nocardia* spp., *Frankia* spp., *Amycolatopsis* spp., and *Actinoplanes* spp. (see Fig. S1 in the supplemental material), and similarly, the classified OBUs from marine samples were all assigned to genomes belonging to the phylum *Actinobacteria*. Canonical analysis of the principal coordinates revealed high levels of OBUs unique to each group (loadings with magnitude > ±0.1); however, none of these OBUs were annotated with homology to KS or AD domains in known genomes in the antiSMASH database or in nucleotide sequences found in NCBI at the time of writing (Fig. 2B).

10.1128/mSystems.00782-20.1 FIG S1 Representation of the relative abundance and distribution of KS and AD domain OBUs with length > 100 bp and E value of <10^−40^ to genomes collected in the antiSMASH database using the nBLAST analysis from soil (DFD_1171.2, DFD_1175.2, DFD_1218.2, and DFD_1305.1), sediment (S_A, S_B, and S_C), and seawater (W_A, W_B, and W_C) samples. (A) Annotated KS OBUs; (B) annotated AD OBUs. Download FIG S1, TIF file, 27.2 MB. Copyright © 2020 Bech et al. 2020 Bech et al. This content is distributed under the terms of the Creative Commons Attribution 4.0 International license.

### Correlation of OBUs and taxonomy.

**FIG 3:**
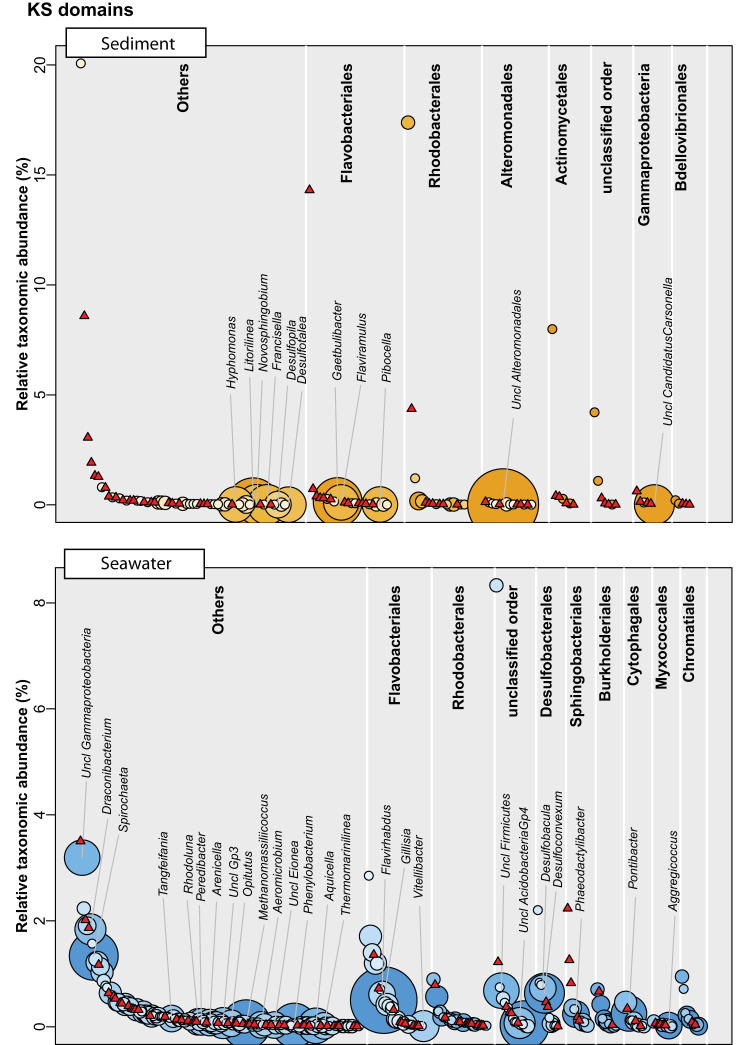
Correlation between genera and KS OBUs in either sandy sediment or seawater (eDNA) samples based on the linear model with *P* value < 0.001. Species are grouped at the genus level and arranged into the top 10 most abundant orders, and the remaining orders are grouped as “Others.” Each genus is plotted according to the relative taxonomic abundance (16S rRNA V3V4 gene diversity) and assigned with the magnitude of the matched KS domain potential (bubbles). Red triangles represent genera with no KS domain correlations. Classified genera with more than five KS OBUs are shown on the plot.

**FIG 4:**
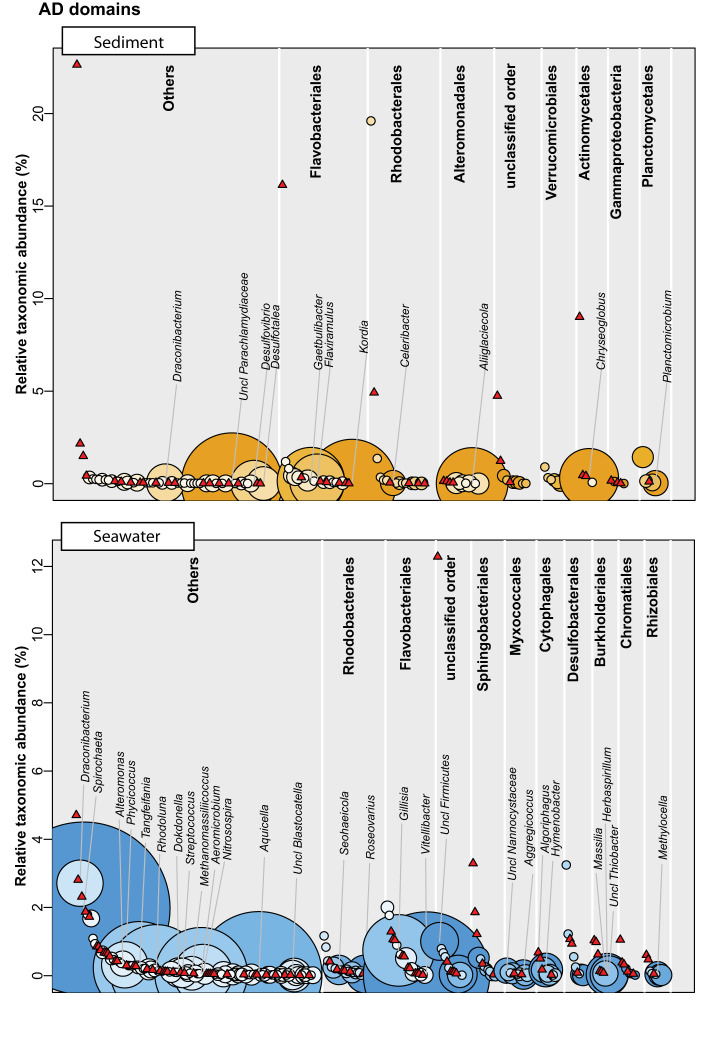
Correlation between genera and AD OBUs in either sandy sediment or seawater (eDNA) samples based on the linear model with *P* value < 0.001. Species are grouped at genera level and arranged into top 9 most abundant orders and the remaining orders as “Others.” Each genus is plotted according to the relative taxonomic abundance (16S rRNA V3V4 gene diversity) and assigned with the magnitude of the matched AD domain potential (bubbles). Red triangles represent genera with no AD domain correlations. Classified genera with more than five AD OBUs are shown on the plot.

Given the poor database-driven annotation rate of the OBUs, we sought to investigate whether they could be coupled to bacterial taxonomy using linear correlations to well-classified ASVs that were assigned to the species level by the BION-meta software. We employed the logic that functional domains are genetically encoded on the same chromosome as the 16S rRNA gene and will cooccur; hence, the two genes will be linearly correlated with one another. This analysis showed that the median values for KS OBUs associated with each species at the genus level were 2 OBUs (range, 0 to 15) in seawater and 1 OBU (range, 0 to 16) in sandy sediments. For AD OBUs, the corresponding values were 1 OBU (range, 0 to 41) in seawater and 2 OBUs (range, 0 to 24) in sediments. In all cases, the distribution of OBUs followed a highly right-skewed distribution with most species having few or zero associated OBUs. For both OBU types and sample types, it was evident that the taxonomic abundance was nonpredictive of the number of OBUs (Fig. 3, Fig. 4; see also Table S1 in the supplemental material), and regression of taxonomic abundance against the number of OBUs revealed an *r*^2^ of ≈0 in all cases.

10.1128/mSystems.00782-20.2 TABLE S1 Number of KS and AD OBUs linked to genera found in seawater and sandy sediment samples based on linear model correlations between the 16S rRNA V3V4 gene and either KS or AD OBU abundances with a *P* value < 0.001 (***). The top five most potential genera for PK and NRP production are listed in the table. Download Table S1, DOCX file, 0.01 MB. Copyright © 2020 Bech et al. 2020 Bech et al. This content is distributed under the terms of the Creative Commons Attribution 4.0 International license.

### Culturable diversity in seawater and sediments.

**FIG 5:**
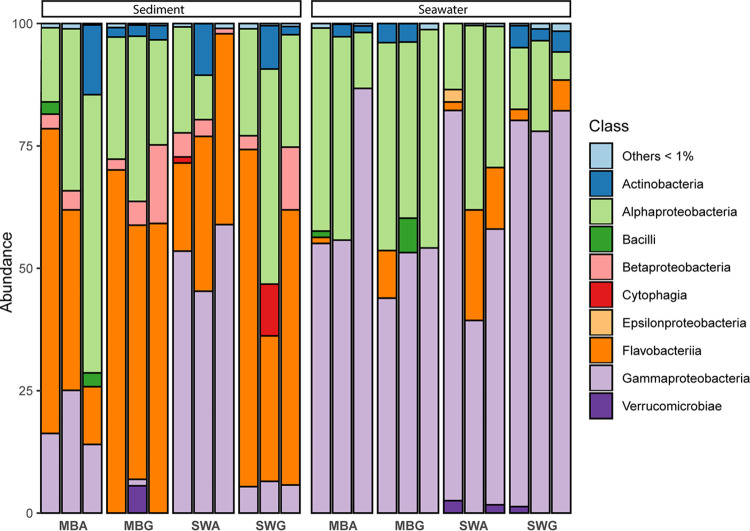
Representation at the class level of the relative taxonomic (16S rRNA V3V4 gene diversity) abundance of the cultured microorganisms (pDNA) isolated from either sandy sediment or seawater. The stacked barplot represents triplicate samples from each growth medium: marine broth agar (MBA), marine broth gellan gum (MBG), seawater agar (SWA), or seawater gellan gum (SWG). Species assigned to orders with <1% of the relative abundance were grouped as Others.

To assess the applicability of conventional culture-based approaches to bioprospecting, microbial communities from seawater and sediments were cultured on different growth substrates with different nutrient compositions and gelling agents. The cultivation approach captured 0.011 to 0.56% and 1.2 to 9.2% of the total microbial cells present in seawater and sediments compared to direct counts using SYBR Gold staining, respectively. An average of 223,943 classified reads were obtained per cultured sample (DNA extracted from the bacterial biomass grown on plates [pDNA]) as summarized in Table 1, representing a total of 762 species. The cultivated community from seawater was dominated by *Alpha*- and *Gammaproteobacteria* of the orders *Rhodobacterales* (median, 29.7%), *Alteromonadales* (median, 12.5%), *Vibrionales* (median, 23.6%), and *Alteromonadales* (median, 12.4%), respectively (Fig. 5). The cultivated sediment samples contained >50% flavobacteria mainly of the order *Flavobacteriales* (median, 45.4%) and a mixture of *Alphaproteobacteria*, *Sphingomonadales* (median, 6.9%), *Rhizobiales* (median, 5.2%), *Rhodobacterales* (median, 4.2%), *Betaproteobacteria*, *Burkholderiales* (median, 3.2%), and *Actinobacteria*, *Actinomycetales* (median, 1.8%; Fig. 5). The culturable taxonomic diversity and microbial plate community structure were not influenced by substrate or gelling agent according to the species richness estimation (*P* = 0.57) and PERMANOVA (*P* = 0.80) analyses. This was mainly due to a high variation between replicates at the species level, yet the agar-based substrates did exhibit a significantly higher proportion of *Gammaproteobacteria* (of different orders) as compared to substrates solidified with gellan gum (*P* = 0.009).

### Biosynthesis potential of culturable bacteria.

**FIG 6:**
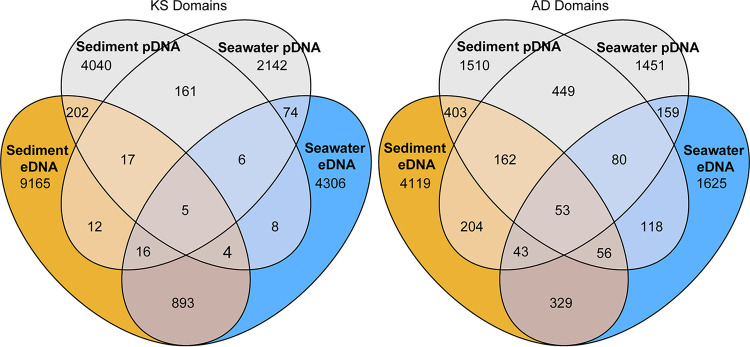
Venn diagrams comparing the recoveries of KS and AD OBUs found in sediment and seawater eDNA in the cultivated fraction. OBUs from the cultivated fraction were pooled depending on the source of isolation (Seawater pDNA and Sediment pDNA).

**FIG 7:**
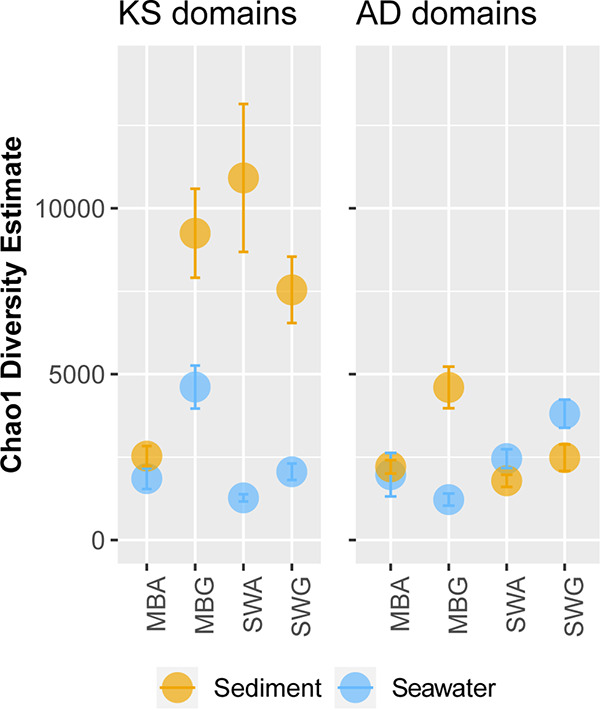
The KS and AD richness in the cultivated fraction (pDNA). Both the KS and AD domain richness were estimated from pooled samples derived from either sandy sediment (yellow dots) or seawater (blue dots). The AD OBUs were further grouped into different growth media (marine broth [MB] or seawater [SW]).

As for the environmental samples, the PKS and NRPS diversity of the cultured fraction was analyzed by amplicon sequencing of the pDNA. A total of 131,838 KS and 217,972 AD reads per sample were retained after clustering at 95% identity on average. A subset of KS and AD OBUs from the eDNA were recovered in the pDNA corresponding to 1.9% of KS and 13.6% of AD OBUs in samples from seawater and 2.2% KS and 12.5% AD OBUs in samples from sediments (Fig. 6). The richness estimates of the cultured samples were highly dependent on the growth conditions (Fig. 7 and Table 1). For KS OBUs, the highest richness overall was found in the sediment-derived samples, in which the highest richness was found when cultured on seawater/agar or marine broth/gellan gum. In the seawater-derived samples, the highest richness was likewise found when cultured on marine broth/gellan gum. For AD OBUs, the overall richness was similar for seawater- and sediment-derived samples, but the highest richness was found in sediment samples cultured on marine broth/gellan gum, which in contrast resulted in the lowest values for seawater-derived samples. For seawater-derived samples, the highest richness was found in seawater/gellan gum cultures. It should be noted that Chao estimates across samples may be overestimated due to the high level of degeneracy in the primers.

**FIG 8:**
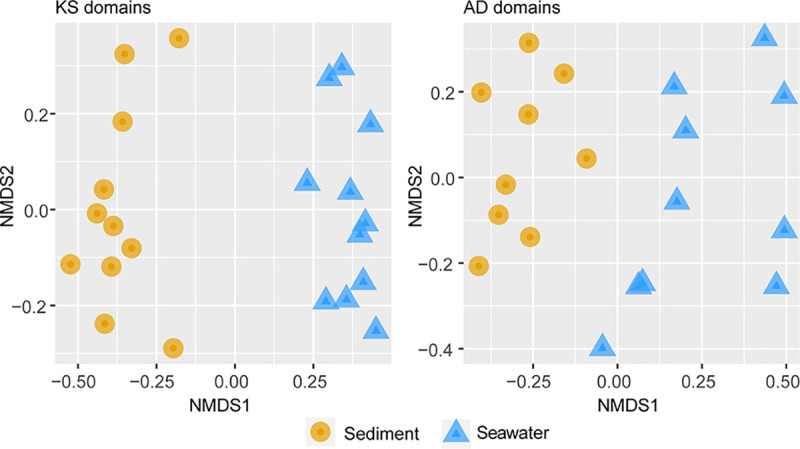
Grouping of the cultivated samples (pDNA) from coastal sediment (yellow circles) and seawater (blue triangles) based on nonmetric multidimensional scaling (NMDS) ordination. Analysis of AD and KS domain based on Bray-Curtis dissimilarities was performed. Differences between the two groups were tested by PERMANOVA (*P* = 0.001).

The overall KS and AD composition was different between cultures derived from seawater and sediments according to multivariate analysis (Fig. 8), which is in agreement with the ordination of eDNA (Fig. 2B).

### Antimicrobial potential in cultured isolates.

In parallel with the extraction of pDNA, we screened a subset of 376 cultured isolates, half from seawater and half from the sandy sediment, for antibacterial activity against a known Gram-negative fish pathogen, Vibrio anguillarum 90-11-286 (29). A total of 33 isolates displayed antimicrobial activity in an initial inhibition assay, and for eight isolates, bioactive compounds could be extracted using ethylacetate. The genomes of these isolates, which represented four members of the *Vibrio* genus, one *Arthrobacter* isolate, one *Pseudoarthrobacter* isolate, one *Aliivibrio* isolate, and one *Pseudoalteromonas* isolate, were sequenced and subsequently subjected to antiSMASH analyses, identifying 31 potential biosynthetic gene clusters; these were mainly identified as NRPS or NRPS/PKS hybrid clusters (Table 2). A total of seven OBUs from seawater and sediment eDNA were recovered in the eight genomes by remapping the OBU amplicons to the genomes. None of these had similarities to previously described clusters. Apart from the *Arthrobacter* and *Pseudoarthtrobacter* genomes which shared 98% average nucleotide identity (ANI), all other genomes had an ANI of less than 90%.

**TABLE 2:** The genetic NP potential of cultivated isolates from coastal seawater and sandy sediment samples with antimicrobial bioactivity analyzed in the antiSMASH database

16S rRNA gene sequence (RDP classifier)	Strain	Source	Medium	No. of contigs	*N*_50_	No. of OBUs from eDNA^*a*^		No. of antiSMASH annotations			
						KS	AD	Siderophore	NRPS	PKS-T1	NRPS/pKS
*Vibrio*	S12_S33	Seawater	MBA	508	75,863	0	0	0	0	1	0
*Aliivibrio*	S10_S31	Seawater	MBA	192	179,121	0	0	1	0	0	0
*Vibrio*	S11_S32	Seawater	MBA	215	75,904	0	0	0	0	0	0
*Vibrio*	S9_S30	Seawater	MBA	255	231,592	0	0	1	7	1	0
*Pseudoarthrobacter*	S13_S34	Seawater	SWA	81	197,152	0	1^*c*^	1	3	0	1
*Pseudoalteromonas*	S16_S37	Seawater	SWG	304	70,525	4^*b*^	0	1	3	0	5
*Vibrio*	S17_S38	Seawater	SWG	66	151,578	0	0	1	0	0	0
*Arthrobacter*	S1_S22	Sediments	MBG	52	275,038	0	2^*d*^	1	3	0	0

a Operational biosynthetic units (OBUs) from the eDNA recovered in the genomes.

b OBU_4427, OBU_8, OBU_4556, and OBU_105.

c OBU_23.

d OBU_42759 and OBU_23.

## DISCUSSION

With the development and refinement of large-scale DNA sequencing technology, profiling of the biosynthesis potential of terrestrial soil microbiomes has intensified in recent years (23, 24, 28). However, much less is known about the potential of microbiomes residing in less intensively studied environments. In this study, we found that the microbial communities in surface seawater and especially in marine sandy sediments are rich in KS and AD OBUs. These OBUs were distinct and different from those found in four representative samples of terrestrial soil microbiomes (23), indicating that novel niches, such as the marine environment, are promising in terms of providing novel NPs that are different from those derived from soil microorganisms. Despite the similar level of taxonomic diversity of the seawater and sandy sediment samples, we found twice the functional diversity in sediments, which suggest that the greater PK and NRP biosynthesis potential is not necessarily associated with the species richness. Interestingly, the estimated richness of both KS and AD OBUs was significantly higher in the marine sandy sediments and for AD OBUs in seawater compared to that of the four soil samples, leading us to investigate which members of the microbial community could be the main drivers of the biosynthesis potential in these environments and whether these were culturable using conventional cultivation approaches.

We used a phenol-chloroform protocol, whereas the soil samples were purified using the DNA Soil Power kit (23). Several studies have found that phenol-chloroform extraction results in higher DNA yields than kit extraction; however, the beta- and alpha-diversity as determined by 16S rRNA gene amplicon sequencing does not differ significantly (30, 31). A direct comparison of the samples is likely to be somewhat occluded by differences in sample preparation, but the large differences in composition observed in Fig. 2A are unlikely to be from extraction efficiency.

The microbial communities of the sediments were fairly similar in composition and rarefaction profile across replicates despite some unevenness in sequencing depth, and the communities were dominated by the *Pelagibacterales* and *Rhodobacterales* of which the SAR11 clade and the species *Planktomarina temerata* were dominating, respectively. The SAR11 clade, including the “*Candidatus* Pelagibacter ubique,” is the most dominating group of bacteria in marine bacterioplankton with a specifically high relative abundance in oligotrophic surface communities, comprising up to 25% of the community (32, 33). “*Candidatus* Pelagibacter ubique” and *Planktomarina temerata* are often cooccurring on the surface of phytoplankton (34), indicating that at the time of sampling, phytoplankton biomass had settled on the surfaces of the sandy sediments. One species relating to Rhodoluna lacicola of the *Actinomycetales* order, which is also often associated with surface freshwater phytoplankton (35), exhibited high relative abundances in the sediment samples as well, corroborating this notion. One hypothesis could be that these community members, which are usually associated with the oligotrophic surface waters, acquire iron by the use of siderophores, containing an AD domain, thus explaining the high AD richness found in the sediment microbiomes. However, neither “*Candidatus* Pelagibacter ubique,” *Planktomarina temerata*, nor Rhodoluna lacicola are known to be prominent secondary metabolite producers. Therefore, other members of the microbial community could contribute to the high biosynthesis potential observed.

To link the taxonomic associations of the NP potential within the marine environments, we used the antiSMASH database to assign the OBUs to high confidence annotated BGCs found in genomes of cultivated bacteria. This analysis showed that more OBUs, particularly from *Actinobacteria*, could be mapped from soil samples compared to samples from seawater and sediments, which could indicate that the marine microbiomes are much less exploited than those of terrestrial systems, at least in our samples. Moreover, historically, the MiBiG database has been based on soil samples, and it is necessarily biased toward this domain. Even the most common KS and AD OBUs found in seawater, sediments, and even in soil samples, had low similarity (<75% identity) to published nucleotide sequences from the MiBiG database or, in a broader scope, the NCBI nr/nt database. Thus, PKs and NRPs that may have a substantial ecological role in the microbial community, and potentially relevant clinical applications, are likely produced by yet uncultured bacteria. Still, culture-dependent methods are considered to be of high importance, especially for understanding the ecological role of secondary metabolites but also due to the many bottlenecks of culture-independent approaches to natural product discovery, such as expression of large BGCs in heterologous host systems. Thus, one promising strategy is to enrich for specific genera of interest and increase the culturability of community members with a diverse secondary metabolism. Previously, such an approach was used for freshwater lake sediments where targeting spore-forming bacteria facilitated the recovery of 7.7 to 23% of PKS type I and II, and NRPSs on agar substrates (36). However, the recovery was estimated using a more conservative threshold (85% identity clustering). We used a fairly short minimum blast length of 100 bp, which might lead to spurious matches. This appears to be less of an issue, since most of the OBUs are not mapped at all. Using a lower cutoff may lead to more mappings, but these may in turn be too spurious to be biologically meaningful or assign taxonomy to.

Since most of the OBUs could not be found in existing databases or in our own isolates, we used a linear model to draw possible links between the bacterial taxonomy and the biosynthesis potential using the 16S rRNA gene amplicons and the KS and AD OBUs. Using this approach, we found that several marine bacteria not normally associated with natural product production were promising as candidates, including *Spirochaetaceae*, *Microbacteriaceae*, and *Alteromonadaceae*. Strikingly, all genera exhibiting a broad biosynthesis potential were low in abundance and constituted at the most ∼0.1% of the total community, which, all things being equal would make them difficult to capture by ordinary bioprospecting approaches. Very recently, other studies have also aimed at linking taxonomic diversity to functionality. Thus, Libis et al. (37) analyzed metagenomic cosmid libraries from soil and subjected them to amplicon sequencing followed by statistical analysis of cooccurrence patterns in order to build complete BGCs. Although the study did not focus on the taxonomic origin of these BGCs, their findings corroborate our findings with respect to the high proportion of BGCs originating from low abundance organisms (37). Borsetto et al. (28) combined 16S rRNA and NRPS/PKS amplicon sequencing and used Mantel and Procrustes tests in the search for overall taxonomic associations at the phylum level. Here, a high NP potential was found in the well-studied *Actinobacteria* and *Proteobacteria*, as well as the less scrutinized phyla *Verrucomicrobia* and *Bacteroidetes* (28). The analysis by Borsetto et al. (28) was not done on lower phylogenetic levels and exclude low-abundant phyla. In an investigation of soil metagenomes, Sharrar et al. (38) generated 1,334 microbial genomes and found a high biosynthesis potential in the phyla *Acidobacteria*, *Verrucomicrobia*, *Actinobacteria*, and *Chloroflexi*, while also noting that the individual members of each phyla often differed greatly in potential. Collectively, these observations suggest that much of the biosynthetic diversity is excluded when omitting low-abundant bacteria, and methods to capture these, such as the iChip (39), are necessary for future NP discovery.

Using different gelling agents in the growth media may facilitate growth of bacterial orders not culturable on agar-based substrates (40). Thus, we used different gelling agents in the present study. We did observe effects on the culturability in terms of bacterial abundance estimates with a specific increase on nutrient-poor substrates, but due to a high variance in the microbial plate community structure between replicates, the influence of different growth substrates on the culturable diversity could not be confirmed. However, agar did facilitate a higher relative abundance and diversity within the *Gammaproteobacteria* from sediment communities. In terms of the efficiency in recovering the biosynthetic diversity present in the marine microbiomes, there was a large if somewhat nonsystematic effect of media and gelling agent, in which gellan gum appeared to be slightly more efficient in capturing both PKs and NRPs. This could be due to the exclusion of certain taxa that are sensitive to the presence of reactive oxygen species formed in agar-containing growth substrates during the sterilization process (41). Interestingly, we found several OBUs exclusively in the pDNA. Presumably, some species exhibit very low abundance in the samples, but due to high culturability, they would be sufficiently abundant for detection in pDNA despite being undetected in eDNA.

Similar to previous observations from freshwater sediments (36), approximately 92% of the OBU diversity from the assessed systems were omitted when culturing, and of the 376 isolates assessed in this study, only 33 showed antimicrobial activity with 8 having extractable antimicrobials. The majority of these were isolated from seawater and were from the families *Vibrionaceae*, *Pseudoalteromonadaceae*, and *Micrococcaceae*, each of which constituted well below 1% of the V3V4 amplicons generated. With this traditional bioscreening approach, in which isolates were selected based on antagonism against a pathogen, we captured only 0.028% of the original diversity. In addition, the highly differentiating OBUs in the ordinations of KS and AD OBUs were not found among the isolates, nor in the pDNA as a whole. Collectively, these results highlight the fact that traditional cultivation approaches are significantly biased toward a small subset of taxa, omitting the majority of the NP biosynthetic genes that could be mined for structural novelty.

### Conclusions.

The marine environment is by far the largest biosphere on the planet, harboring a multitude of diverse microbial niches. Compared to their terrestrial counterparts, these niches have in large part been neglected in the search for novel natural products. In our sequencing-based approach to assessing the biosynthesis potential of marine microbiomes, we have demonstrated that the richness of NP biosynthesis genes may rival that of soils, suggesting that coastal seawater and especially marine sandy sediment likely are promising troves of novel chemistry. Linear regression analyses were used to estimate possible links between taxonomy and biosynthetic potential, and they indicated that a high biosynthetic repertoire could be harbored in low abundance taxa. Classical cultivation techniques failed to capture the majority of the biosynthesis diversity observed, hence, targeted cultivation regimes are needed to facilitate the recovery of novel bioactive compounds produced by marine bacteria in the future.

## MATERIALS AND METHODS

### Sampling and bacterial abundance estimates.

All samples were collected outside Hellerup harbor, Denmark (55°73′18′’, 12°58′27′’) on 10 August 2017 at 9:30 a.m. (18.5°C, 0.5 mg liter^−1^ dissolved oxygen [DO], 18.39 [practical salinity units {PSU}]). Seawater samples were collected at approximately 0.1-m depth, and sediment cores were collected from the upper surface layer of the sandy sediment outside the harbor. Biological triplicates were collected approximately 1 m apart. Total number of bacterial cells were determined using SYBR Gold and previously described epifluorescence microscopy methods (42). For seawater samples, undiluted and 10-fold-diluted samples were counted, and for sediment samples, 5 g of sediment was suspended in 45 ml 2% Instant Ocean (IO; Aquarium Systems Inc., Sarrebourg, France) followed by bacterial detachment with sonication in a 2 × 50 W sonication bath at 28 kHz for 4 min. Detached cells were subsequently filtered and counted. Culturability was estimated by the fraction of CFU/milliliter relative to the SYBR Gold counts.

### Growth media and cultivation conditions.

Processing and plating of the samples were done as described previously (40). Dilution series of the triplicate seawater samples were plated directly onto four different substrates, and cells from triplicate sediment samples were plated following detachment as described above. The four substrates contained Marine Broth (MB) (Difco catalog no. 2216) or seawater (SW) solidified with agar (MBA or SWA) or gellan gum (CP Kelco, San Diego, CA, USA) (MBG or SWG) (40). Plates were incubated at 25°C for 2 weeks (MB) or 5 weeks (seawater). Forty-seven randomly picked colonies from each growth substrate were transferred to cryoprotectant solution and stored at −80°C.

### DNA extractions from seawater, sediments, plate DNA, and bacterial isolates.

Initially, cell lysates were prepared as follows for all sample types. Environmental DNA (eDNA) from particle-associated and planktonic bacteria in seawater was extracted by filtering a total volume of 300 ml seawater through a 0.2-μm polycarbonate filter. Subsequently, the filters were submerged in 1 ml lysis buffer (400 mM NaCl, 750 mM sucrose, 20 mM EDTA, and 50 mM Tris-HCl [pH 8.3]). Sediment samples were prepared by mixing 1 g of sediment with 1 ml lysis buffer. Plate DNA (pDNA) from the cultivated fraction was obtained from three replicate plates containing approximately 200 separable macrocolonies each. Biomass was harvested by washing the plates with 1 ml lysis buffer. Lysate from bioactive isolates was prepared using 2-ml overnight culture by suspending a cell pellet (centrifugation at 8,000 × *g* for 5 min) in 1 ml of lysis buffer.

The continued DNA extraction procedure was adapted from the procedure in reference 43 and applied to all the lysates described above. Lysozyme (Sigma, St. Louis, MO, USA) was added to a final concentration of 1 mg ml^−1^, and samples were incubated at 37°C for 30 min. Sodium dodecyl sulfate (SDS) and proteinase K (Sigma, St. Louis, MO, USA) were added to final concentrations of 1% and 0.1 mg ml^−1^, respectively. After incubation overnight at 55°C with regular agitation, samples were centrifuged at 3,000 × *g* for 5 min, and the supernatants were transferred to new tubes. Phenol-chloroform-isoamyl alcohol (25:24:1 [vol/vol/vol]; Sigma, St. Louis, MO, USA) was added to an equal amount of volume to each sample, followed by an extraction of the aqueous phase with 1 volume of chloroform-isoamyl alcohol (24:1 [vol/vol]; Sigma, St. Louis, MO, USA). The DNA in the aqueous phase was precipitated by adding 0.1 volume of 3 M Na-acetate [pH 5.5] and 0.6 volume of ice-cold isopropanol followed by an additional washing step in 96% ethanol. The air-dried DNA pellets were dissolved in DNase-free water.

### Amplicon sequencing of the 16S rRNA V3V4 region.

The V3V4 region was amplified using each eDNA sample and DNA from the cultivated fraction (pDNA) as the template in the subsequent PCR applying primers tagged with octameric barcodes (see Table S2 in the supplemental material) in PCRs containing 10.6 μl DNase-free water, 12.5 μl TEMPase Hot Start 2× Master Mix, 0.8 μl of each forward (Fw_V3V4; 5′-CCTACGGGNGGCWGCAG-3′) and reverse (Rv_V3V4; 5′-GACTACHVGGGTATCTAATCC-3′) primer (10 μM) and 0.3 μl DNA template with the amplification conditions of 95°C for 15 min, followed by 30 cycles, with 1 cycle consisting of 95°C for 30 s, 62°C for 30 s, and 72°C for 30 s, and a final step of 72°C for 5 min. All V3V4 amplicons were cleaned using Agencourt Ampure XP magnetic beads (0.6:1 bead volume to DNA solution) pooled in equimolar ratios (50 ng per amplicon). Amplicons were submitted to the Center for Biosustainability, Technical University of Denmark (DTU), Denmark for sequencing on an Illumina MiSeq 300PE platform.

10.1128/mSystems.00782-20.3 TABLE S2 Barcodes used for amplicon sequencing of the 16S rRNA gene as well as OBUs of biosynthetic gene clusters in eDNA isolated from seawater and sandy sediment samples and marine microorganisms cultured on different substrates. Each barcode was used for each sample across V3V4, AD, and KS primers. Since there were more samples than barcodes, each barcode was used twice by further multiplexing with Illumina adaptors. Download Table S2, DOCX file, 0.01 MB. Copyright © 2020 Bech et al. 2020 Bech et al. This content is distributed under the terms of the Creative Commons Attribution 4.0 International license.

### Amplicon sequencing of the KS and AD domains.

Degenerate primer pairs targeting NRP adenylation (AD) domains (A3F0X [5′-GCSTACSYSATSTACACSTCSGG] and A7R0X [5′-SASGTCVCCSGTSCGGTA]) and PK ketosynthase (KS) domains (degKS2F0Y [5′-GCNATGGAYCCNCARCARMGNVT] and degKS2R0Y [5′-GTNCCNGTNCCRTGNSCYTCNAC]) were used to PCR amplify biosynthetic domains from each eDNA and pDNA sample in the first PCR round. Primers also contained the invariant Illumina p5 or p7 sequence, an 8-bp barcode sequence (Table S2) and a spacer sequence (F0X and R0X) as described previously (23). Amplicons from the first round were used as the template DNA in a second PCR round with the following primers: MiSeq Forward: 5′-CAAGCAGAAGACGGCATACGAGATGTGACTGGAGTTCAGACGTGTGCTCTTCCGATCT-3′; MiSeq Reverse: 5′-AATGATACGGCGACCACCGAGATCTACACTCTTTCCCTACACGACGCTCTTCCGATCT-3′.

The amplification of the AD domains was set up in 25-μl PCR mixtures as follows: 9.9 μl DNase-free water, 12.5 μl TEMPase Hot Start 2× Master Mix (VWR International, Søborg, Denmark), 0.8 μl A3F0X (10 μM), 0.8 μl A7R0X (10 μM), and 1 μl DNA. Amplification conditions for AD domain primers were as follows: 95°C for 15 min, followed by 45 cycles, with 1 cycle consisting of 95°C for 30 s, 56°C for 30 s, and 72°C for 45 s, and a final step of 72°C for 10 min. Amplification conditions for KS domain primers were as follows: in 25-μl PCR mixture, 7.2 μl DNase-free water, 12.5 μl TEMPase Hot Start 2× Master Mix, 2.4 μl degKS2F0Y (20 μM), 2.4 μl degKSR0Y (20 μM), and 1 μl DNA. The amplification conditions for KS domain primers were as follows: 95°C for 15 min, followed by 45 cycles, with 1 cycle consisting of 95°C for 30 s, 55°C for 30 s, and 72°C for 45 s, and a final step of 72°C for 10 min. In a second PCR round, the 50-μl PCR mixtures were set up as follows: 20.8 μl DNase-free water, 25 μl TEMPase Hot Start 2× Master Mix, 1.6 μl of each MiSeq forward and reverse primers (10 μM), and 1 μl PCR template. Amplification conditions for MiSeq primers were as follows: 95°C for 15 min, followed by 20 cycles, with 1 cycle consisting of 95°C for 30 s, 56°C for 30 s, and 72°C for 20 s, and a final step of 72°C for 5 min. All amplicons from the second round were cleaned using Agencourt Ampure XP magnetic beads (0.6:1 bead volume to DNA solution; Beckman Coulter, USA), pooled in equimolar ratios (2 μg per sample). Amplicons were submitted to BGI Tech Solutions, Hong Kong, for sequencing on an Illumina MiSeq 300PE platform.

### Processing the V3V4 amplicons.

Sequence reads from each samples were demultiplexed, cleaned, chimera filtered, divided into ASVs, which were classified to species level with the RDP-II SSU database using the software BION-meta (Danish Genome Institute, Aarhus, Denmark). The combined species table was constructed with Phyloseq R package (44). Sequences classified to chloroplasts were excluded.

### Processing KS and AD domain sequences.

The fastq files of the raw KS and AD amplicon sequences were analyzed according to Charlop-Powers et al. (23). The forward and reverse reads were first demultiplexed into sample specific fastq files. The fastq files were debarcoded with cutadapt (45) and quality filtered using the trimfq command in seqtk (https://github.com/lh3/seqtk). Forward and reverse reads were trimmed to 240 bp and 175 bp, respectively, and paired-end reads were joined together with a single “N” spacer between the forward read and the reverse complement of the second read with USEARCH32 (46). The joined reads were within-sample clustered at 97%, *de novo* chimera checked with VSEARCH (47), pooled in KS and AS pools, respectively, and clustered in a second round at 95%. All remaining singletons were discarded from the final pools. Reads in the final 95% identity clusters were used to construct a combined table with operational biosynthetic units (OBUs) in Phyloseq (44). The complete data set of raw fastq files of KS and AD domains found in urban park soils from Charlop-Powers et al. (23) were kindly provided by Zachary Charlop-Powers. Of the 275 soil samples that were collected and analyzed across the five boroughs of New York City, NY, in Charlop-Powers et al., we selected four randomly picked fastq forward and reverse pair files from the list “Dataset_S01” file of reference 23. The four samples, “DFD_1171.2,” “DFD_1175.2,” “DFD_1218.1,” and “DFD_1305.1” were sampled at Allay Park, Alley Park, Clove Lakes Park, and Blooming Dale Park, respectively. All four samples represented an average richness (Chao1 estimates) of the KS and AD diversity calculated in Charlop-Powers’ study.

The amplicon sequences from the four soil samples were processed through the same pipeline described above for comparison of the NP potential in soil microbiomes to that found in seawater and sediment microbiomes collected in this study.

### Richness, composition, and statistical analysis.

Rarefaction of the KS and AD OBUs and 16S rRNA V3V4 species reads were generated in R version 3.5.1. The Chao1 richness estimates were used to predict the alpha-diversity (44) and tested with *t* test or analysis of variance (ANOVA) followed by Tukey’s test if significant. Subsequently, all samples were normalized to 100,000 reads to compare the KS, AD, and species composition between marine seawater, sandy sediments, and soil. Multivariate patterns in the eDNA and pDNA samples were visualized by principal coordinate analysis (PCoA) and nonmetric multidimensional scaling (NMDS), respectively, and tested for group-wise differences with PERMANOVA from the R package vegan, all using Bray-Curtis distances. Canonical analysis of principal coordinates was used to evaluate OBUs or species of interest.

### Linking functional OBUs to taxonomy.

In order to correlate the OBUs to the classified ASVs that were assigned to species level within each niche, all matrices were cleaned, e.g., OBUs or species found in only one sample and with a total read size of <10 were removed. Internal Pearson correlation coefficients within the species table were calculated in R version 3.5.1, where species with an *r*^2^ of 1 were removed. Subsequently, linear regression models without intercepts were fitted according to the equation

$${\text{OBU}}_{i}=\alpha_{i,j}{\;\text{species}}_{j}$$

where OBU*_i_* is the *i*th functional domain, species*_j_* is the *j*th species, and *α_I_*_,_*_j_* is the slope coefficient of the association of OBUs and species found in each group, respectively. Linear models (LM) that did not have a *P* value of < .001 were discarded. The final result was a list of species with correlating OBUs visualized in bubble plot at the genus level rank ordered by abundance versus the numeric abundance, stratified by order. Each point was furthermore expanded as a function of number of associated OBUs.

### Assessing antibacterial activity of cultured bacterial isolates.

Antibacterial activity of a total of 376 isolates was assessed isolating at random 47 strains from each of the four growth media from each of the two sample types. Antibacterial activity was determined against the fish pathogen Vibrio anguillarum 90-11-287 (29). The pathogen was inoculated in half-strength YTSS (1/2YTSS; 0.2% yeast extract, 0.125% tryptone, 2% Sigma sea salts) broth, incubated overnight at 25°C with agitation (200 rpm), and subsequently embedded into either of the four media used in the original cultivation procedure (MBA, MBG, SWA, or SWG) at a 5,000 × dilution equivalent to approximately 10^5^ CFU/ml. Isolates from MB-based plates were precultured in liquid MB, and isolates from SW-based plates were precultured in half-strength MB (1/2MB) medium for 24 h at 25°C with agitation (100 rpm). Five microliters of overnight culture was spotted on the corresponding growth medium from which they were isolated with the embedded V. anguillarum strain 90-11-287. Thirty-three isolates produced inhibition zones against V. anguillarum. These isolates were grown in 50 ml MB or 1/2MB for 48 h at 25°C, and 20 ml of the outgrown cultures was extracted with equal volumes of ethyl acetate. The organic phase was collected and evaporated under N_2_ flow at 37°C, redissolved in 200 μl methanol, and tested for antibacterial activity in a well diffusion assay.

### Whole-genome sequencing, assembly, and identification of bioactive isolates.

Fifty nanograms of genomic DNA (gDNA) from the isolates was sequenced on the Illumina MiSeq 300PE platform at the Center for Biosustainability, DTU, Denmark. The genomes were assembled by SPADES 3.13 (48). The isolate taxonomy was determined by blastn of the 16S rRNA gene against the NCBI nt database. Genomes were investigated for BGCs using antiSMASH 5 (3). Genomes were compared using average nucleotide identity (ANI) using ANIm with the pyani package (49).

### OBU blastn annotations.

The OBUs in all the samples were annotated by a blastn using a custom nucleotide database of PKS-KS and AD domains extracted from the antiSMASH database version 2 (50) with an E-value cutoff < 10^−40^ and a minimum length of 100 bp. Because the inserted “N” was incompatible with blastn, only the forward reads was used. To remap OBUs to isolates, all OBUs sequenced from the eDNA were subjected to a BLAST search against the assembled genomes with an E-value cutoff of <10^−40^, a minimum length of 100 bp, and sharing more than 97% identity.

### Data availability.

Sequencing data are available in the Sequencing Read Archive (SRA), amplicon data in bioproject PRJNA613816, and WGS data in bioproject PRJNA613817.
