# Supplementary figures and images for: Marine Sediments Hold an Untapped Potential for Novel Taxonomic and Bioactive Bacterial Diversity
Source: mSystems. 2020 Sep 15;5(5):e00782-20. doi: 10.1128/mSystems.00782-20 (PMC7498687; doi:10.1128/mSystems.00782-20)

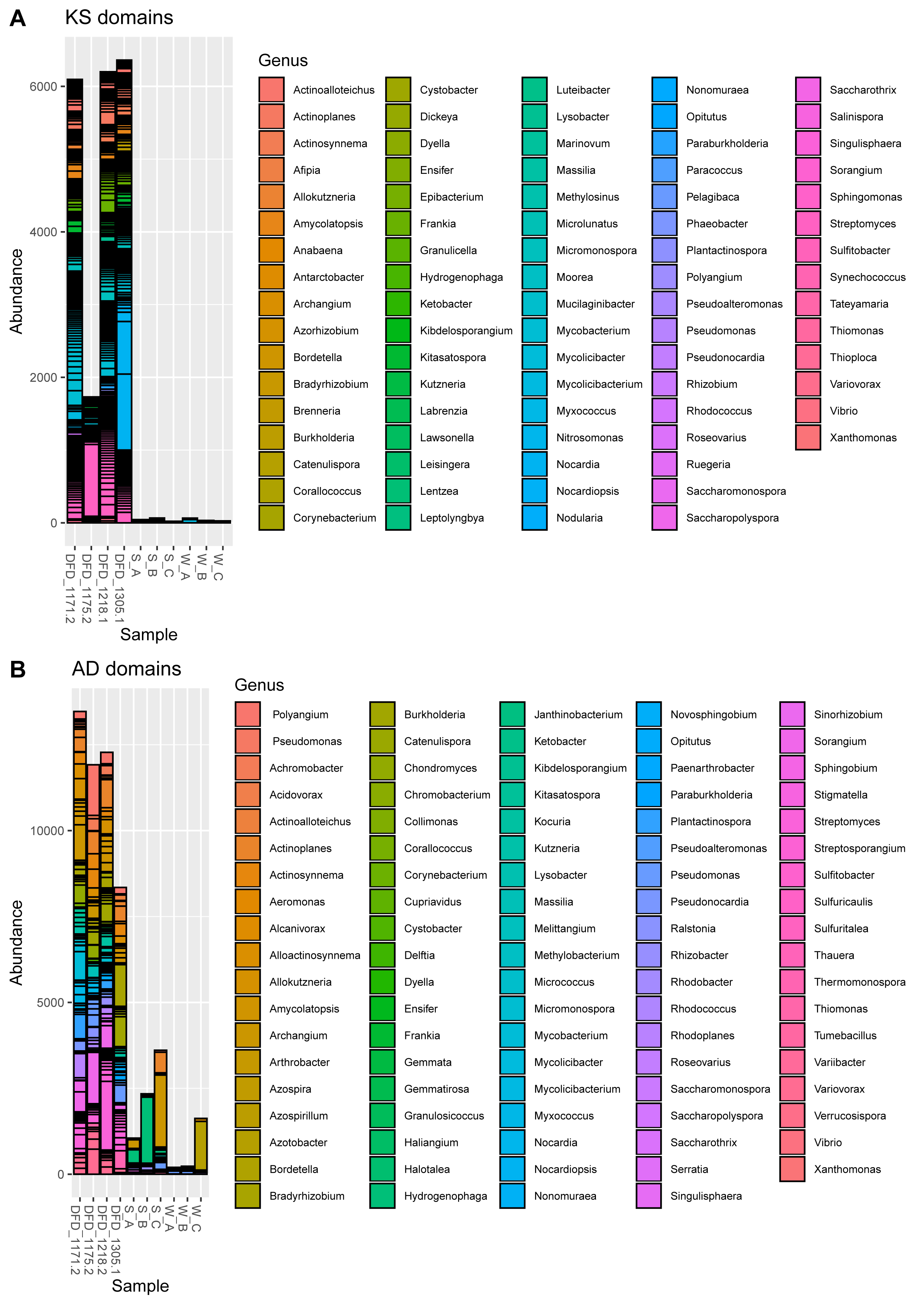

Supplement: FIG S1 [file mSystems.00782-20-sf001.tif]
