# Supplementary material for: Marine Sediments Hold an Untapped Potential for Novel Taxonomic and Bioactive Bacterial Diversity
Source: mSystems. 2020 Sep 15;5(5):e00782-20. doi: 10.1128/mSystems.00782-20 (PMC7498687; doi:10.1128/mSystems.00782-20)
Supplement: TABLE S1 [file mSystems.00782-20-st001.docx]

| Domain | Sample | Phylum | Order | Family | Genus | #OTUs | Relaetive taxonomic abbundance (% %) | #OBUs |
| --- | --- | --- | --- | --- | --- | --- | --- | --- |
| KS | Sediment | Proteobacteria | Alteromonadales | unclassified | unclassified | 1 | 0,02 | 16 |
|  |  | Chloroflexi | Caldilineales | Caldilineaceae | *Litorilinea* | 2 | 0,01 | 12 |
|  |  | Bacteroidetes | Flavobacteriales | Flavobacteriaceae | *Gaetbulibacter* | 4 | 0,12 | 11 |
|  |  | Proteobacteria | Sphingomonadales | Sphingomonadaceae | *Novosphingobium* | 14 | 0,01 | 9 |
|  |  | Proteobacteria | Thiotrichales | Francisellaceae | *Francisella* | 4 | 0,01 | 9 |
|  | Water | Bacteroidetes | Flavobacteriales | Flavobacteriaceae | *Gillisia* | 5 | 0,45 | 15 |
|  |  | Spirochaetes | Spirochaetales | Spirochaetaceae | *Spirochaeta* | 5 | 1,19 | 11 |
|  |  | Euryarchaeota | Methanomassiliicoccales | Methanomassiliicoccaceae | *Methanomassiliicoccus* | 1 | 0,04 | 11 |
|  |  | Proteobacteria | Caulobacterales | Caulobacteraceae | *Phenylobacterium* | 8 | 0,02 | 10 |
|  |  | Proteobacteria | Desulfobacterales | Desulfobacteraceae | *Desulfoconvexum* | 1 | 0,56 | 9 |
| AD | Sediment | Chlamydiae | Chlamydiales | Parachlamydiaceae | unclassified | 1 | 0,01 | 24 |
|  |  | Bacteroidetes | Flavobacteriales | Flavobacteriaceae | *Kordia* | 2 | 0,01 | 21 |
|  |  | Proteobacteria | Alteromonadales | Alteromonadaceae | *Aliiglaciecola* | 3 | 0,01 | 17 |
|  |  | Bacteroidetes | Flavobacteriales | Flavobacteriaceae | *Gaetbulibacter* | 4 | 0,12 | 16 |
|  |  | Actinobacteria | Actinomycetales | Microbacteriaceae | *Chryseoglobus* | 1 | 0,25 | 14 |
|  | Water | Spirochaetes | Spirochaetales | Spirochaetaceae | *Spirochaeta* | 5 | 0,12 | 41 |
|  |  | Proteobacteria | Legionellales | Coxiellaceae | *Aquicella* | 3 | 0,12 | 30 |
|  |  | Bacteroidetes | Flavobacteriales | Flavobacteriaceae | *Vitellibacter* | 2 | 0,07 | 30 |
|  |  | Bacteroidetes | Bacteroidales | Prolixibacteraceae | *Tangfeifania* | 1 | 0,06 | 22 |
|  |  | Actinobacteria | Actinomycetales | Microbacteriaceae | *Rhodoluna* | 2 | 0,00 | 22 |
