# Supplementary material for: Marine Sediments Hold an Untapped Potential for Novel Taxonomic and Bioactive Bacterial Diversity
Source: mSystems. 2020 Sep 15;5(5):e00782-20. doi: 10.1128/mSystems.00782-20 (PMC7498687; doi:10.1128/mSystems.00782-20)
Supplement: TABLE S2 [file mSystems.00782-20-st002.docx]

| # | Barcode |
| --- | --- |
| 1 | TTTTAATC |
| 2 | ATAATTAG |
| 3 | ACCAAATT |
| 4 | CTTATCAA |
| 5 | TGATCATT |
| 6 | AGAATCTA |
| 7 | TCAAGAAA |
| 8 | ATCGAAAT |
| 9 | ACATTTAC |
| 10 | TAGAAAAC |
| 11 | TTATCACC |
| 12 | AATAGGGT |
| 13 | ATTGCTGA |
| 14 | TGAGTTCT |
| 15 | GGCTATTT |
| 16 | CAAGAGAT |
| 17 | GGAATACA |
| 18 | AAGGCAAT |
| 19 | ACAAAACG |
| 20 | TTGAGTGA |
| 21 | GCTTCTGA |
| 22 | GGCAAGAT |
| 23 | GTGCTTTC |
| 24 | ACACACTG |
| 25 | CGATTCTG |
| 26 | GCAGAGTT |
| 27 | CGTCCTAT |
